# 11.H. Round table: Timely access to quality-assured medicines worldwide

**DOI:** 10.1093/eurpub/ckac129.716

**Published:** 2022-10-25

**Authors:** Organised by: EUPHA-HTA, EMA, WHO, Chair persons: Elena Petelos (EUPHA-HTA), Dineke Zeegers Paget (EUPHA)

## Abstract

A key aspect of achieving UHC and reducing health inequity is ensuring the timely access to high-quality, safe and effective medicines across the world. The regulatory approval of such products in countries with limited regulatory resources can be lengthy, often compromising patients’ timely access to much-needed medicines. This resource-demanding process extends well beyond marketing authorisation, incl. for post-approval changes. The WHO Collaborative Registration Procedure uses the Stringent Regulatory Authorities’ medicines evaluations (SRA CRP), a procedure that allows National Regulatory Authorities (NRAs) to leverage the work performed by Stringent Regulatory Authorities (SRAs) on scientific evaluations to decide on medical products approvals within their jurisdiction, through the concept of reliance. Reliance allows an authority to leverage the work performed by other authorities, such as scientific evaluations, to decide on medical products approval within their jurisdiction. This reduces duplication of regulatory efforts, resources and time, while maintaining national sovereignty. In the context of these processes, multi-agency partnerships and cross-border collaboration the role of European bodies, and specifically that of the European Medicines Agency (EMA) as a Stringent Regulatory Authority will be explored. The SRA CRP and its pilot has been evaluated, based on data and stakeholders’ perceptions. The evaluation indicates that the procedure delivers on expected objectives and benefits, including shortened timelines (from submission to approval), reduced duplication of efforts and resources (human and financial). It also shows significant and long-term positive impact for the LMICs involved. Additional benefits include greater application of international harmonized standards, an added capacity-building component and an informed and high-quality decision-making at the NRA. The impact of the procedure can be seen through 59 approvals for 16 medicines in 23 countries. The key areas for improvement and recommendations include greater collaboration, communication and transparency between NRAs, applicants, SRAs and WHO, to facilitate interactions and regular sharing of information between stakeholders. It is key to optimise resource utilisation and to leverage synergies, and to this end, a centralized, live platform could be developed to share the status, progress and challenges of applications in real time including information related to post-approval changes. This panel will present the SRA CRP pilots and its evaluation, bringing together experts from regulatory agencies and multilateral bodies to elucidate the current degree and extent of collaboration, whilst attempting to role of the EU and of the EMA in these processes. The panelists will also have an opportunity to take questions from the audience and to discuss the contribution of the EU to global health, incl., timely access for people across the world.

**Key messages:**

• The SRA CRP facilitates and accelerates the regulatory approval of quality-assured, safe and effective medicines by reliance on SRAs, while maintaining decision-making national sovereignty.

• The EU, via the EMA and the EUM4all and SRA CRP mechanisms, promotes international collaboration and reliance pathways for earlier access to medicines and contributing to global public health and UHC.

**Speakers/Panellists:**

**Victoria Palmi**

European Medicines Agency, Amsterdam, Netherlands

**Mariana Roldão Santos**

WHO, Geneve, Switzerland

**Richard Pilsudski**

Sanofi, Paris, France

